# Volume growth trend and correlation of atrial diameter with lateral ventricular volume in normal fetus and fetus with ventriculomegaly: A STROBE compliant article

**DOI:** 10.1097/MD.0000000000016118

**Published:** 2019-06-28

**Authors:** Hong-Li Ma, Si-Xiu Zhao, Fu-Rong Lv, Zhi-Wei Zhang, Yun-Hua Xiao, Bo Sheng

**Affiliations:** a Department of Radiology, First Affiliated Hospital of Chongqing Medical University, Yuanjiagang, Chongqing; b Department of Radiology, People's Hospital of Chongqing Yubei District, China.

**Keywords:** 3D MRH, fetal magnetic resonance imaging, growth trend, lateral ventricular volume

## Abstract

To explore the growth trend of fetal lateral ventricular volume, for understanding the relationship between atrial diameter (AD) and volume in normal fetus and fetus with ventriculomegaly.

Overall, 97 sequential fetal head magnetic resonance imaging scans were performed; these pertained to 50 fetuses with normal lateral ventricles [normal group; gestational age (GA): 24–38 weeks] and 47 fetuses with ventriculomegaly (VM) (VM group; GA: 24–37 weeks). The left, right, and total lateral ventricular volume were measured using 3-dimensional magnetic resonance hydrography (MRH). Correlation coefficient (r) was calculated to assess the relationships of measurements. Lineal regression analysis was used to assess correlation of AD and GA with volume. Between-group differences in terms of AD and volume were assessed using *t* test.

Significant linear growth was observed in the total lateral ventricular volume compared with GA in the normal group with a relative growth rate of 2.87% per week (*P* <.001). Significant linear relationship between AD and volume was observed, and a significant equation was acquired in the normal group and VM groups, respectively, using the simple linear regression model: left volume = 0.438 ∗ normal left diameter (NLD) + 1.359; right volume = 0.493 ∗ normal right diameter (NRD) + 1.012; left volume = 0.959 ∗ left diameter in VM (VLD) − 2.074; right volume = 0.799 ∗ right diameter in VM (VRD) − 0.443. A significant equation was obtained in the normal group and the VM group, using the multiple linear regression model: Total volume (mL) = 0.396 ∗ NLD + 0.410 ∗ NRD + 3.101; and total volume = 0.989 ∗ VLD + 0.834 ∗ VRD − 3.141, respectively. In terms of AD and volume, the left lateral ventricle was significantly larger than the right side in both groups. The volume of lateral ventricle in AD ≥10 mm group was larger than that in the AD <10 mm group. The total volume in the VM group was significantly larger than that in the normal group.

The total lateral ventricular volume increased with GA. AD can be used to evaluate the fetal ventricular volume.

## 1 Introduction

Monitoring of brain growth during the intrauterine period is clinically relevant. The fetal lateral ventricles (LVs) can be used as an anatomical reference to locate the brain and determine the transverse sections during the extremely early stages of pregnancy; this is because the fetal LVs are the easiest to detect among the fetal brain structures using sonography.^[1]^ The ventricular atrial diameter (AD) can be used to predict the function of the fetal nervous system.^[2]^ The normal lateral ventricular diameter at the level of the atrium is <10 mm by sonography; ≥10 mm diameter can be diagnosed as ventriculomegaly (VM) at any gestational age (GA).^[3,4]^ The severity of VM is categorized as mild (10–12 mm), moderate (12–15 mm), and severe (>15 mm).^[5,6]^ VM can be caused by various of disorders that lead to poor outcomes, such as neurological, motor, and/or cognitive impairment.^[7,8]^

Therefore, it is crucial to accurately measure the LV, for the diagnosis of VM, as well as for its role as an indicator of associated fetal anomalies to determine whether further investigation is warranted, and for classification purposes in clinical research. The LV is routinely measured at the level of the atrium, which may fail to comprehensively portray the dynamic changes in size and/or morphology of the LV because it solely represents the characteristics of the posterior aspect.^[9]^ However, the volume measurement of LV can compensate for the above limitations and can be used to predict etiology and prognosis in fetuses with VM.^[10]^ Our study aimed to describe volume growth trend and to assess the relationship between AD and volume.

The volumetric measurement method 3-dimensional magnetic resonance hydrography (3D MRH) was used in this study to measure the volume of the LV; this method is commonly used in cholangiopancreatography.^[11,12]^ Besides, 3D MRH facilitates the allows for visualization of slowly flowing or stationary fluids in high-signal density structures, against a dark background with very low signal density.^[13]^

## 2 Material and methods

### 2.1 Subjects

This was a single-center prospective study conducted at our hospital from March 2017 to April 2018. Ethical approval was granted by the Ethics committee of the First Affiliated Hospital of Chongqing Medical University and the ethical clearance number is 20192901. Written informed consent was obtained from all the pregnant women. The diagnosis of VM was determined on ultrasound and magnetic resonance imaging (MRI; unilateral or bilateral LVs ≥10 mm). The inclusion criteria were single fetus with VM and normal LVs. The exclusion criteria were: twin pregnancy, poor image quality, and unstable clinical condition. A total of 105 fetuses were enrolled and assessed during the study period. Eight patients were excluded (6 because of poor image quality and 2 were excluded because of unstable clinical condition). Finally, data pertaining to 97 fetuses were included in the analysis. The GA was evaluated from the first trimester dating ultrasound scan.

### 2.2 Equipment and methods

All the fetal brain MRI examinations were performed using a 1.5T scanner (GE Healthcare, Milwaukee, WI) with an 8-channel full-body coil. Sedation was not required and scan time was ≤45 minutes. Patients were instructed to breathe calmly and try to keep themselves as still as possible in a supine position during the scan. 3D fast-recovery-fast-spin echo (FRFSE) with the array spatial sensitivity encoding technique was performed in the axial plane of the fetal brain using the following parameters: repetition time (TR)/echo time (TE) = 1700 ms/500.5 to 508 ms; bandwidth = 62.50 Hz; slice thickness = 2 mm; matrix = 288 × 224; field of view (FOV) = 34 cm × 34 cm; sequence acquisition time = 40 to 55 seconds; number of slices = 65 to 81; and with fat saturation. To avoid folding artifacts, FOV was moderately broadened in the slice direction. Second or third scans were performed if the imaging quality was unqualified because of fetal motion. We performed T2-weighted single-shot fast-spin echo (SSFSE) in the coronal, sagittal, and axial planes of fetal brain with the following parameters: TR/TE = 2000 ms/140 ms; slice thickness = 3 mm; intersection slice gap = 0; matrix = 256 × 192; FOV = 38 × 38 cm; sequence acquisition time = 29 to 45 seconds; number of slices = 12 to 15.SSFSE images were obtained in an interlaced manner for reducing the saturation of spins in adjacent slices and were solely designed for clinical scan. Each sequence served as a positioning criterion for subsequent sequences.

### 2.3 Linear and volumetric analysis

**Figure 1:**
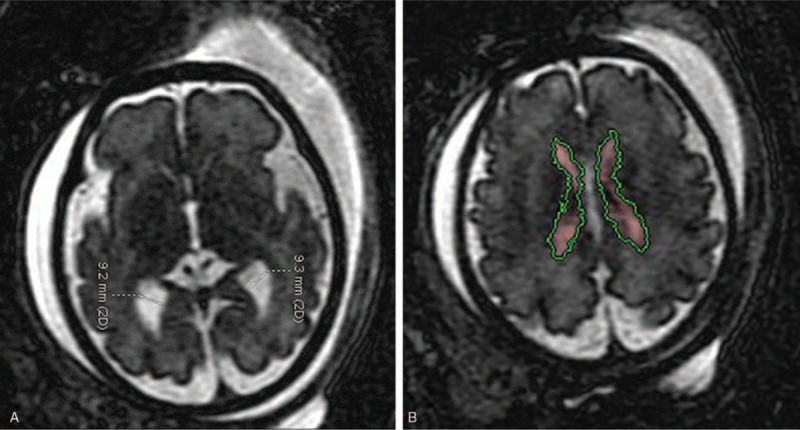
Linear (A) and volumetric (B) measurement of lateral ventricle in axial plane.The calipers were placed on border of ventricular wall and perpendicular to the long axis of the ventricle (A). The software semiautomatically segmented the lateral ventricles on the strength of image contrast which originated from user-defined thresholds (B).

**Figure 2:**
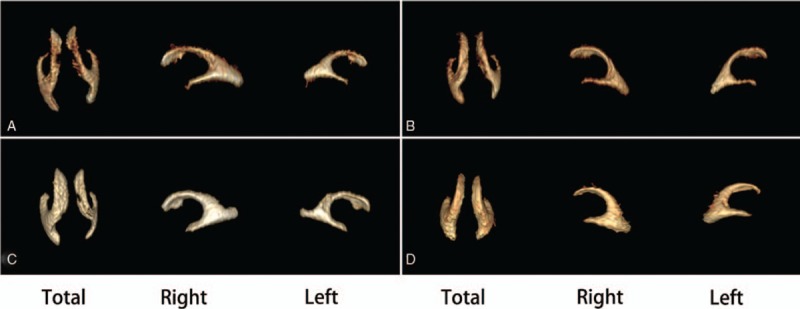
Total, right and left volume reconstructed by 3D MRH. A normal fetus [Total volume (TV):11.995 mL, Right volume (RV): 7.173 mL, Left volume (LV): 4.822 mL] aged 34+2 weeks (A);A normal fetus [TV:10.168 mL, RV: 5.648 mL, LV: 4.511 mL] aged 33 weeks (B); A fetus [TV:15.027 mL, RV: 8.933 mL, LV: 6.094 mL] aged 31+5 weeks with unilateral ventriculomegaly (C); A fetus [TV:19.081 mL, RV:9.705 mL, LV: 9.376 mL] aged 32+4 with bilateral ventriculomegaly (D).

Measurement of the LV diameter was conducted at the standard axial plane which displayed both the posterior and frontal horns with the choroid plexus being clearly visible (Fig. 1). Measurement of the volume of LV acquisition was performed using Advantage Windows Volume Viewer Software (v.11.3, GE Healthcare) in an Advantage Windows Workstation (AW 4.6, GE Healthcare). 3D FRFSE sequence images were processed using a post-processing method, which involved semi-automatic image segmentation via multiple volume reformation with volume rendering technology (Fig. 2). Our previously published article describes the volume reconstruction in detail.^[14]^ The total lateral ventricular volume, as well as the volume of each lateral ventricle, were measured and recorded.

### 2.4 Statistical analysis

The IBM SPSS (Version 22, IBM, Armonk, NY) was used to perform the data analyses. Data pertaining to LVs are presented as mean ± standard deviation or median (range) depending on data distribution. Spearman's rank correlation coefficient (r) was used to assess the strength of the relationship between measurements and GA. Pearson correlation coefficient was calculated to assess the relationship between AD and volume. Simple linear regression analysis and multiple linear regression model were used to assess the correlation of AD and GA with volume. Paired *t* test was used to perform the comparison of left and right AD and volume; an independent samples *t* test was used to assess between-group differences in terms of the total ventricular volume. *P* <.05 was considered statistically significant. Relative growth rate per week was calculated by dividing growth per week by the average volume.

## 3 Results

### 3.1 Subjects

A total of 97 cases were included in the study: 50 in the normal group [median age: 33.1 weeks (range, 24–38.7)] and 47 in the VM group [mean age: 31.5 ± 2.6 weeks (range, 24–37)]. In the VM group, 34 fetuses were unilateral and 13 fetuses were bilateral.

### 3.2 Normal group

**Figure 3:**
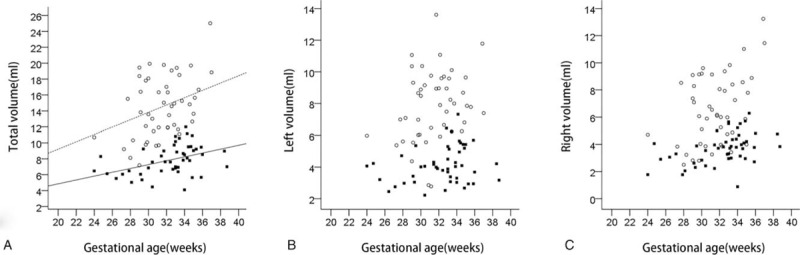
The scattergrams of the linear regression analysis between total (A), left (A), right (C) lateral ventricular volume and gestational age. The volumes corresponding to gestational age in normal fetus (solid squares) and fetus with ventriculomegaly (open circles) are showed. The relationships between gestational age and the volume of total lateral ventricle in normal fetus (solid line) and fetus with ventriculomegaly (dotted line) are provided.Total volume in the VM group was significantly larger than that in the normal group (t = −10.803, *P* <.001).

The range of AD, lateral ventricular volume and total lateral ventricle volume were 1.9 to 9.5 mm, 0.89 to 7.32 mL, and 4.1 to 12 mL, respectively (Table 1). Right and total lateral ventricular volume showed a significant moderate correlation with GA [0.49 (*P* <.001) and 0.43 (*P* = .002), respectively] (Fig. 3). A slight growth in total volume was observed at a relative growth rate of 2.87% per week.

**Table 1:**
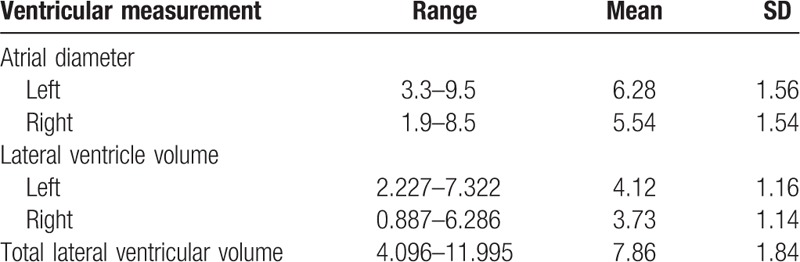
Measurements of atrial diameter (mm) and volume (mL) in the normal lateral ventricle.

**Figure 4:**
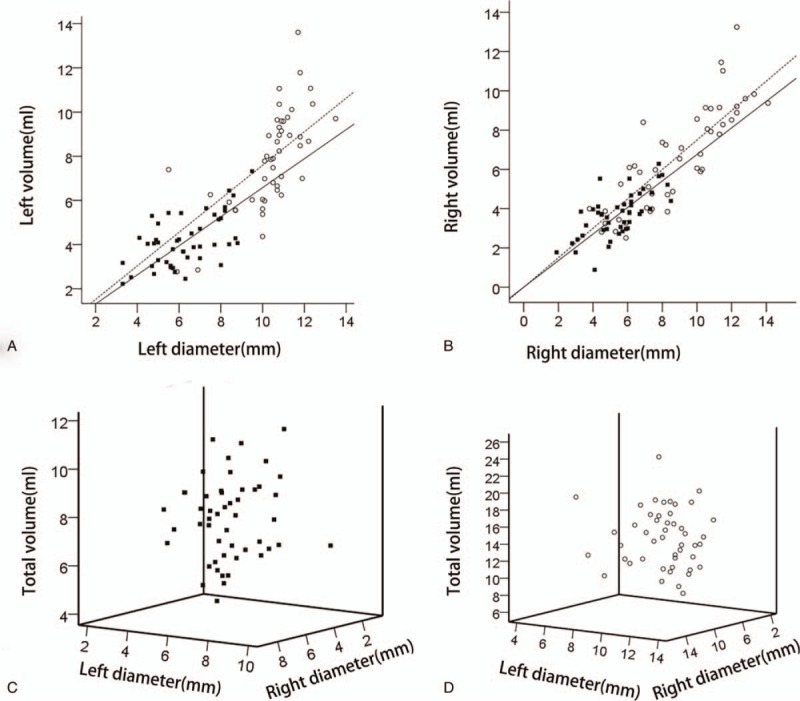
The scattergrams of lineal regression analysis between atrial diameter and volume. The volumes corresponding to ipsilateral diameter or total volume corresponding to bilateral diameters in normal fetus (solid squares) and fetus with ventriculomegaly (open circles) are showed. The relationships between left diameter and left lateral ventricular volume (A), right diameter and right lateral ventricular volume (B) in normal fetus (solid line) and fetus with ventriculomegaly (dotted line) are showed. The scattergrams involving in the relationships among total volume, right diameter, left diameter in normal fetus (C) and fetus with ventriculomegaly (D) are described.

Simple linear regression model was used to determine the linear relationship between AD and volume of each LV. Normal left diameter (NLD) was found to account for 33.9% of left volume variability on the basis of the adjusted R-squared (R2adj) value obtained with the equation: left volume = 0.438 ∗ NLD + 1.359 (*P* <.001).Normal right diameter (NRD) was found to account for 45.4% of right volume variability the equation was as follows: right volume = 0.493 ∗ ALD + 1.012 (*P* <.001). The left side was significantly greater than the right side in terms of LV diameter and volume (t = 3.045, *P* = .004; and t = 2.076, *P* = .043, respectively) (Fig. 4).

NLD and NRD cumulatively could significantly account for 28% of total volume variability using the multiple linear regression model with the following equation: total volume = 0.396 ∗ NLD + 0.410 ∗ NRD + 3.101 (*P* = .011 for NLD, *P* = .008 for NRD, F = 10.524, *P* <.001, R2adj = 0.28) (Fig. 4).

### 3.3 VM group

Total lateral ventricular volume in the VM group was significantly larger than that in the normal group (t = −10.803, *P* <.001). The mean ± standard deviation of right AD as well as the left, right, and total lateral ventricular volumes were 8.83 ± 2.23 mm, 7.89 ± 2.21 mL, 6.62 ± 2.57 mL, and 14.50 ± 3.83 mL, respectively. The median left diameter in VM (VLD) was 8.6 mm (range, 3.8–14.1 mm). Right and total lateral ventricular volumes showed a significant correlation with GA [Pearson correlation coefficient: 0.43 (*P* = .003) and 0.41 (*P* = .004), respectively] (Fig. 3).

**Figure 5:**
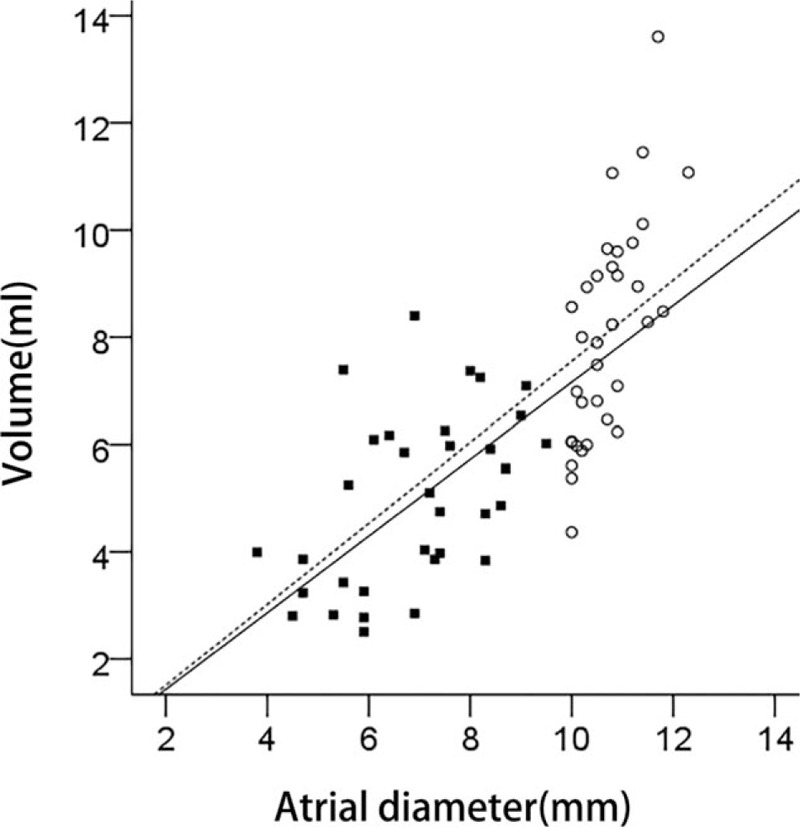
Lineal regression analysis between atrial diameter and volume AD <10 mm group (solid squares, solid line) and AD ≥10 mm group (open circles, dotted line). AD = atrial diameter.

A significant equation was obtained using the simple linear regression model: left volume = 0.959 ∗ VLD – 2.074. VLD was found to account for 12% of left volume variability. Right diameter in VM (VRD) accounted for 69.2% of right volume variability with the equation: right volume = 0.799 ∗ VRD − 0.443 (*P* <.001) (Fig. 4). Overall, 34 fetuses exhibited unilateral VM; these were divided into 2 groups: AD <10 mm (6.96 ± 1.48, range 3.8–9.5) and AD ≥10 mm. AD showed a significant correlation with volume in the 2 groups (r = 0.49, *P* = .003 for the AD <10 mm group; r = 0.724, *P* <.001 for the AD ≥10 mm group) (Fig. 5). Lateral ventricular volume in the AD ≥10 mm group was greater than that in the AD <10 mm group.

VLD and VRD cumulatively accounted for 45.6% of total volume variability using the multiple linear regression model with the following equation: total volume = 0.989 ∗ VLD + 0.834 ∗ VRD − 3.141 (*P* = .001 for VLD, *P* <.001 for VRD; F = 20.283, *P* <.001) (Fig. 4).

## 4 Discussion

Although ultrasound (US) is the standard imaging modality used for the diagnosis of fetal VM, it is vulnerable to the effects of fetal position, maternal obesity, presence of oligohydramnios, field of view, operator's skills, and acoustic shadowing from the ossifying calvarium.^[15,16]^ With the advent and development of ultrafast acquisition sequences, fetal MRI can overcome the above limitations because of its ability to capture image in more than 1 plane as well as to provide higher contrast resolution, and better tissue characterisation.^[17,18]^ In addition, it is more effective than US for the detection of congenital malformations, thereby rendering MRI a complimentary diagnostic method to assess equivocal findings, to improve diagnostic confidence, to elucidate additional pathology that is not visualized on sonography, and to guide pre- and postnatal treatment and parental counseling for future pregnancies.^[19,20]^

The 3D MRI was used in the present study because of the following advantages:

(1) a more comprehensive delineation of structural and morphological changes is possible as it facilitates reformatting into any standard plane;
(2) the decreased slice thickness improves spatial resolution and enables the observation of subtle changes;
(3) the reconstructed volume image can vividly depict changes in morphology by rotating and cutting compared with 2D. Moreover, 3D MRI can be better applied to the third trimester than 3D US.

Furthermore, 3D reconstruction and manual multisection planimetry (MSP) has been the most common methods employed for volumetric measurement in recent studies;^[21–23]^ however, these are time-consuming and technically challenging methods. With the same capabilities as MSP, 3D MRH requires less time (4 min VS12 min) and contains excellent inter-rater agreement and intra-rater repeatability.^[14]^

In our study, the total volume in the normal group was larger than that reported from in previous study.^[24]^ The differences may be attributable to race, heterogeneous cohorts, and different methodology. In our previous study, the volume obtained using 3D MRH was shown to be slightly greater than that obtained using MSP^[14]^.

During the normal development of the fetal LV, it remains relatively stable in terms of the size of the fetal LV from 14 weeks onwards up to birth.^[25]^ Significant small linear growth was observed in the total lateral ventricular volume from 24 to 37 weeks in the normal group with a relative growth rate of 2.87% per week. Previous studies of lateral ventricular volume have yielded variable results in this respect including growth,^[24]^ reduction^[26]^ and stabilization.^[22]^ A study showed a small increase with a relative growth rate of 3.43% per week which was slightly higher than that observed in the present study.^[24]^ Heterogeneous cohorts and the inclusion of third ventricle or cavum septum pellucidum may explain the differences among the studies.

The measurement of the LV diameter is routinely recommended during fetal scans performed during the second trimester for screening of fetal anomalies; therefore, we chose the AD as a linear index and explored its relationship with volume. Left atrial diameter (LAD) and right atrial diameter (RAD) were researched in the light of their independent correlation with the volume of each LV and total lateral ventricular volume in the normal and VM group. LAD and RAD accounted for 30.9% and 48% of the total volume variation in the normal and VM groups. Our findings in terms of the relationship between AD and volume in VM group are consistent with those of a previous study.^[27]^ The equations acquired in the present study suggest that the lateral ventricular volume could be estimated using AD. To the best of our knowledge, this is the first study to evaluate the association between linear measurement and volume of the normal LV.

In the present study, total lateral ventricular volume in the VM group was significantly greater than that in the normal group. Our results are consistent with those of previous studies. Lateral ventricular volume in the AD ≥10 mm group was greater than that in the AD <10 mm group, as anticipated.

One of the limitations of our study is the relatively small sample size in both groups. This may limit the statistical analysis. Another limitation is the uneven data distribution pertaining to the GA. We did not include the data before GA as well as that after GA of 37 weeks. Moreover, lesser data are available on border GA and some have only 1 or 2 cases. Therefore, a study with a larger volume of data and uniform distribution of GA is required.

## 5 Conclusion

In the present study, we confirmed that the total lateral ventricular volume increased with GA and that ventricular volume can be evaluated by AD.

## Acknowledgments

We gratefully acknowledge the help from Dr Ke Shi of Department of Radiology, West China Hospital, Sichuan University, Sichuan, China.

## Author contributions

**Conceptualization:** Hong-Li Ma, Zhi-Wei Zhang, Yun-Hua Xiao.

**Data curation:** Hong-Li Ma, Si-Xiu Zhao, Bo Sheng.

**Formal analysis:** Hong-Li Ma, Bo Sheng.

**Investigation:** Hong-Li Ma.

**Methodology:** Hong-Li Ma, Si-Xiu Zhao, Zhi-Wei Zhang, Yun-Hua Xiao.

**Software:** Si-Xiu Zhao.

**Supervision:** Fu Rong Lv.

**Validation:** Fu Rong Lv, Yun-Hua Xiao.

**Visualization:** Fu Rong Lv.

**Writing – original draft:** Hong-Li Ma.

**Writing – review & editing:** Hong-Li Ma, Fu Rong Lv.

Fu rong Lv orcid: 0000-0001-9301-7474.
